# Ionisation

**Published:** 1911-03-18

**Authors:** 


### IONISATION.

It is a long time since men began to use the chemical action of a galvanic current to introduce remedies into the body without the intervention of the stomach and assmilating organs.

Ulcers of the leg were treated by a very simple apparatus: a plate of sheet zinc of the same size and shape as the sore was connected by a copper wire with a small plate of thin silver. The epidermis was removed from a patch of skin near the ulcer by means of a blistering plaster, and the silver plate was secured by a bandage to the raw surface. It will be seen that the amount of electrolytic action in this clever circuit will be limited by the area of the silver plate. The whole application was kept on for several days at a time.

The "Apostoli" treatment of uterine fibroids, which was successful in the hands of many surgeons, probably owed its influence to the action of copper carried by electrolytic action from the metal stilette into the substance of the tumour.

About three years ago Dr. Lewis Jones, of St. Bartholomew's Hospital, proposed the use of solution of zinc sulphate as an application to rodent ulcers. He has had marked success with the same method as that advocated by Dr. Leduc. A continuous battery of about ten cells is connected with a zinc plate electrode large enough to cover the sore. The strength of current required varies according to the area of the sore; 2 milliampères per square centimetre can be borne for fifteen to twenty minutes, supposing that the zinc sulphate solution is of not more than 2 per cent. strength. This is applied in several layers of gauze laid on the sore and gently pressed by the zinc electrode. Other surgeons have had excellent results from this treatment of common and erythematous lupus, epithelioma, and large flat warts.

The writer has had good results from potassium bromide electrolysed in the skin over the scars left by herpes zoster. The pain of the persistent neuritis, very difficult to control by any ordinary means, is much relieved by this application. A zinc electrode covered with flannel soaked in 10 per cent. bromide solution is to be applied for twenty minutes every day. Some men prefer an electrode which consists of a bell-shaped glass whose area is similar to the commonly used circular-plate zinc electrode. It is filled with solution of the usual strength, retained in the glass by a porous diaphragm of flannel stretched across the mouth. It is evident that the dosage of the drug to be conveyed, and of the area of the electrode, must be adapted to the case under treatment.

Ionisation may also be effected by immersing the part affected in the solution of bromide or other drug, the battery being connected up in the usual way.

It is not essential that all drugs used in this way should be dissolved. Salicylates have been ionised through the skin of the leg and arm, first powdering them freely with the drug. The drug is moistened by the warm water in the linen or other covering of the electrode, which is to be made large enough to reach from hip to ankle or shoulder to hand. Thin zinc makes a good cathode because it can be bent to the shape of the limb affected. The application should last for thirty minutes for each limb treated thus, and should be used daily. The continuous current in medicine has not been used often enough or long enough at a time.

Salicylate of soda is used by Wullyamoz, of Lausanne, in subacute cases of rheumatism. A patient who had suffered severely on three previous occasions from acute rheumatism had taken salicylates by the mouth for six to twelve weeks at a time, with little benefit. After five sittings of half an hour each, the applications being used every other day, swelling and pain were so far relieved that the patient could walk about and could sleep well.

Other more acute cases yielded to similar treatment when salicylate had been taken for several days without success.

The current used with these large electrodes was from 160-200 milliampères, and is said to have caused no pain. The loss of heat was unusually rapid, 102° to 98° in twenty-four hours.

Ophthalmia in new-born or young children has been easily removed by using a pure zinc wire, or with a thin layer of cottonwool as anode or positive pole. A very weak current and a short application are needed. Of course cocaine is required. Similar treatment cures corneal ulcers. When it is desired to ionise iodine, as for chronic arthritis, solution of iodine is painted over the joint, which is then covered by a flexible electrode, attached to the negative pole of the battery, and covered with flannel or lint, dipped in warm water. If iodide of potassium or sodium is to be used, it is dissolved in the water used to moisten the lint. The strength of such solution is 1 to 2 per cent., and the milliampères in the circuit should be at first ten, gradually increased to thirty or forty, in the course of half as many minutes.

Dr. Lewis Jones had had much success in treating large joints by this method. He says that the pain and vesication which are obstacles to this treatment may be obviated by using several layers of linen or cotton cloth soaked in the solution.

In the case of open sores, considerable pain may be caused by ionisation, which cocaine does not always relieve, and a general anæsthetic has been required. Professor Leduc, of Nantes, says that he has not failed in obtaining relief in any arthritic case he has ionised with iodine. A patient whose right fingers were ankylosed, some months after she came under his care was playing tennis with the same hand. Carefully raised, a current of 40 milliampères was borne after fifteen minutes.

The apparatus required is very simple. Schall offers a suitable battery of twenty small Leclanché cells, and to this a milliampère meter should be attached. Some dial current selectors have the disadvantage that the cells 1 to 10 are always in use, and cells 20 to 30 are much less used.

Electrodes of zinc of various areas complete the outfit.

